# The impact of pricing strategy on the costs of oral anti‐cancer drugs

**DOI:** 10.1002/cam4.2269

**Published:** 2019-05-27

**Authors:** Judy Truong, Kelvin K. W. Chan, Helen Mai, Alexandra Chambers, Mona Sabharwal, Maureen E. Trudeau, Matthew C. Cheung

**Affiliations:** ^1^ Department of Medicine University of Toronto Toronto Canada; ^2^ Division of Hematology and Oncology Odette Cancer Centre, Sunnybrook Health Sciences Centre Toronto Canada; ^3^ Pan‐Canadian Oncology Drug Review Canadian Agency for Drugs and Technologies in Health Toronto Canada; ^4^ Division of Biostatistics, Dalla Lana School of Public Health University of Toronto Toronto Canada; ^5^ Canadian Centre for Applied Research in Cancer Control Vancouver Canada

**Keywords:** anti‐cancer (anticancer) drugs, anti‐cancer (anticancer) medications, cancer drug price(s), cost analysis, health economics, pricing strategy

## Abstract

**Background:**

The soaring costs of anti‐cancer drugs pose a threat to the sustainability of cancer care. The pricing strategy chosen by manufacturers can impact the costs of oral anti‐cancer drugs during dose modifications, but this issue remains under‐recognized in the literature. In general, with the flat pricing strategy, there is a single fixed price for each tablet regardless of dosage strength, whereas with linear pricing, the price of each tablet increases with its dose. We hypothesize that flat pricing will have increased drug costs compared to linear pricing during dose reductions since the cost remains fixed despite decreased dose requirements. This practice may have significant financial implications considering the high costs, extensive utilization, and frequent occurence of dose reductions with anti‐cancer drugs.

**Methods:**

Oral anti‐cancer drugs reviewed by the pan‐Canadian Oncology Drug Review program between 2011 and 2018 were identified. The cost per mg and cost per 28‐day cycle were calculated for dose levels −2 to +2. The percent change in cost per mg and cost per cycle during dose modifications from the standard dose were calculated. We conducted Mann‐Whitney *U* and Fisher‐exact tests to compare the association between drug costs during dose reductions and pricing strategy.

**Results:**

In this study, 30 oral anti‐cancer drugs for use in 41 indications were analyzed; 44% of drugs used linear pricing and 56% used flat pricing. Dose reductions increased the mean cost per mg for drugs with linear pricing by 14.7% (range: 0%‐50%) at dose level −1 and 17.2% (range: 0%‐50%) at dose level −2 and flat pricing by 60.8% (range: 19%‐100%) at dose level −1 and 99.1% (range: 0%‐300%) at dose level −2. The cost per mg was significantly increased in drugs using flat pricing compared to linear pricing when dose reduction to either level ‐1 (*P* = 0.010) or level ‐2 (*P* = 0.006) occurred. The mean cost per cycle was decreased for drugs using linear pricing by 20.9% (range: −40% to 0%) at dose level −1 and 48.7% (range: −60% to −25%) at dose level −2 and flat pricing by 0.8% (range −6% to 0%) at dose level −1 and 11.0% (range: −50% to 100%) at dose level −2. The cost per cycle was significantly decreased in drugs with linear pricing compared to flat pricing when the standard dose is reduced to either dose level ‐1 (*P* = 0.005) or dose level ‐2 (*P* = 0.026).

**Conclusions:**

Overall, flat pricing had significantly greater costs compared to linear pricing during dose reductions of anti‐cancer drugs. While there is a general expectation that the cost of drugs should decrease with dose reduction, drugs with flat pricing were generally found to have increased cost per mg and no change in the cost per cycle. The resultant increased spending on drug acquisition (despite purchasing lower doses) lead to financial wastage, which has significant implications on cost‐effectiveness considerations and budgets. Future economic evaluations should take into consideration the hidden costs associated with dose reductions of flat priced drugs.

## 1 INTRODUCTION

The rising cost of cancer care is unsustainable. It is projected to increase by 27% to more than $158 billion (USD) from 2010 to 2020.^1^ A recent study showed that the economic burden of cancer care in Canada more than doubled from $2.9 billion in 2005 to $7.5 billion in 2012; chemotherapy, in particular, accounted for one of the largest increases (by a factor of 3) in expenditures.^2^ Within the pharmaceutical market, oncology drugs are the therapeutic class with the greatest global spending.^3^ Novel oral anti‐cancer drugs routinely cost more than $100,000 (USD) per year.^4^ The rising utilization of oral anti‐cancer drugs has raised concerns about their affordability due to out‐of‐pocket expenses, which shift the financial burden onto patients.^5^ Financial toxicity has been increasingly recognized as an adverse effect of cancer treatment.^5^, ^6^ Financial barriers may lead to poor adherence to medications, resulting in worse health outcomes, including earlier mortality.^7^ Over the past decade, novel anti‐cancer drugs have had increasing costs associated with higher launch prices and subsequent post‐launch price elevations without a proportional increase in their clinical benefits.^8^, ^9^, ^10^, ^11^, ^12^, ^13^, ^14^, ^15^ These prohibitive costs have prompted the need to critically evaluate the value of anti‐cancer drugs. Several organizations, including the American Society of Clinical Oncology^16^ and the European Society for Medical Oncology,^17^ have developed frameworks to appraise the value of anti‐cancer agents, which includes and assessment of the clinical benefits, toxicities, and costs of these medications.^18^

In Canada, new anti‐cancer drugs are initially reviewed by Health Canada for safety and efficacy to be approved for use. In order for the drug to be eligible for public reimbursement, the pan‐Canadian Oncology Drug Review (pCODR) within the Canadian Agency for Drugs and Technologies in Health (CADTH), independently reviews the evidence to provide recommendations to the provinces.^19^ There are three possible recommendations: (a) reimburse, (b) reimburse with clinical criteria and/or conditions, (c) do not reimburse.^20^ The provinces, however, do not have to accept the recommendations from pCODR. As such, there can be variability in drug funding between provinces. The assessment includes an evaluation of the clinical effectiveness, cost‐effectiveness, feasibility of adoption, and patient values. The pharmacoeconomic data reviewed by the pCODR include cost‐effectiveness and budget impact analyses. However, current guidelines for conducting economic evaluations do not provide guidance on how to address the potential impact of pricing strategy.^21^, ^22^, ^23^, ^24^

Pricing strategy can potentially modify the cost‐effectiveness and budget impact of a drug. In general, there are two pricing strategies: linear (or monotonic) and flat pricing.^25^, ^26^ Linear pricing sets the price of each tablet or capsule such that the price increases with its dosage strength, but this does not necessarily have to be in a proportional manner. In contrast, with flat pricing, the price of the drug is the same regardless of dosage strength. Flat pricing is advantageous in enabling prescribers the ability to select the optimal dose without concern for increased costs and it offers better predictability of expenditures for financial planning. However, there is a potential for the flat pricing strategy to increase costs during dose reductions. For example with ruxolitinib, the price is fixed at the same rate regardless of its strength (available in 5 mg, 15 mg, and 20 mg). When ruxolitinib was first approved for myelofibrosis by the pCODR, the 10 mg tablet was not yet available. An individual with myelofibrosis would require one 15 mg tablet at the standard dose, but if the individual required a dose reduction (from 15 mg to 10 mg), two 5 mg tablets would be required. Due to flat pricing, the individual would incur twice the cost for drug acquisition despite requiring a lower dose. Since most oral anti‐cancer drugs are covered under provincial drug plans, the increased costs will predominantly affect provincial budgets, but there will be some additional out‐of‐pocket costs to the individuals. The impact of this practice on a societal level is currently unknown.

Since anti‐cancer drugs are costly and dose reductions are common in this setting,^27^ the potential impact of these costs could be significant. Furthermore, groups that are more prone to require dose reductions, such as the elderly, may be unfairly disadvantaged due to this pricing practice. Given the paucity of attention of this issue in the literature, we investigated the impact of pricing strategy on the costs of anti‐cancer drugs during dose modifications. We compared the drug costs (cost per mg and cost per 28 days) between linear and flat pricing. We hypothesize that flat pricing will result in increased drug costs during dose reductions compared to linear pricing. Understanding of the impact of pricing strategy on economic evaluations may help policymakers and payers make better decisions about drug coverage and resource allocation under constrained budgets.

## 2 METHODS

### 2.1 Oral Anti‐Cancer drugs

Oral anti‐cancer drugs with Health Canada approval and submitted to the pCODR with “notified to implement” review status were identified between July 2011 (inception of the pCODR) and January 2018 from the CADTH website. The status “notified to implement” means that the review of the drug submission is complete and that the final recommendations on whether the drug product should be publicly reimbursed is available. We did not include drug submissions that were withdrawn, incomplete, or under review. The most recently approved drug submission for each indication was used to obtain the generic name, brand name, indication(s), publicly available drug price(s), dosage form (capsule or tablet), dosage strength(s), and the recommended starting dose.

Dose reduction or escalation is considered when optimizing the effectiveness and toxicities of a drug. The drug monograph specifies when dose modification is indicated and provides the recommended dose for that dose level. Dose level 0 refers to the standard dose. Dose level modification is step‐wise; the dose at level 0 is greater than at level −1, which in turn is greater than the dose at level −2. Similarly, dose level +1 is greater than dose level 0, and dose level +2 is greater than +1. However, dose level +2 does not necessarily mean twice the recommended dose; the specific dose at each level is obtained from the drug monographs. Usually only two dose level modifications are allowed before the drug is discontinued. The dosing for dose levels −2 to +2 was obtained from drug monographs and/or the Cancer Care Ontario Drug Formulary.

### 2.2 Pricing strategy

The pricing strategy (linear or flat) was identified for each drug with multiple dosage strengths. The pricing strategy was determined based on the steepness of pricing. This was calculated using the methodology of Jönsson et. al.,^25^ where the difference in price between the highest and lowest strength of the drug was divided by the difference in strength. This price ratio was then divided by the price per mg for the lowest strength to normalize it to the lowest strength. The greater the price ratio, the steeper the pricing strategy used. A ratio of 0 represents perfect flat pricing and a ratio of 1 represents perfect monotonic or linear pricing (see Appendix S1 for sample calculations). Three categories were identified by Jönsson et. al^25^: 0‐0.33 (flat pricing), 0.34‐0.65 (intermediate), 0.66‐1 (linear pricing). In our study, flat pricing will refer to drugs that have a relatively fixed price per tablet or capsule regardless of dosage strength defined by a price ratio less than 0.34. Linear pricing will refer to drugs where the price of a tablet or capsule increases with its dosage strength in a relatively proportional manner defined by a price ratio greater than 0.65. Drugs with intermediate price ratios do not have a predominant pricing strategy.

### 2.3 Analysis

The impact of oral drug pricing strategies on costs was determined for dose modifications between dose levels −2 and +2 for each drug indication if possible. The estimated drug costs per mg and costs per 28‐day cycle were calculated for the following scenarios where possible: (a) standard dose, (b) dose reductions to dose level −1 and −2, and (c) dose escalations to dose level +1 and +2. The cost per mg was calculated by dividing the price of the tablet or capsule by the mg. The overall drug cost per 28 days was calculated using the minimum combination of tablets or capsules to achieve the required dose multiplied by the number of times taken per day and the number of days per cycle, normalized to a 28‐day cycle. The percent change in cost per mg and cost per cycle due to dose modifications in comparison to the standard dose scenario (dose level 0) were calculated. The percent change in cost per mg and cost per 28 days during dose modifications from the standard dose were compared between drugs using flat and linear pricing.

We conducted Mann‐Whitney *U* and Fisher‐exact tests to compare the association between costs during dose reductions and pricing strategy. The Mann‐Whitney *U* test was used to compare the percent change in cost per mg and cost per cycle during dose reductions between drugs with flat or linear pricing. Fisher‐exact tests were performed to compare the number of drugs that had an increase in the cost per mg to those that did not during dose reductions. We also performed Fisher‐exact tests to compare the number of drugs that had a decrease in the cost per cycle compared to those that did not during dose reductions between flat and linear pricing. All *P*‐values < 0.05 were considered significant. All statistical analyses were performed using R version 3.5.1.

## 3 RESULTS

### 3.1 Oral Anti‐Cancer drugs

**Figure 1:**
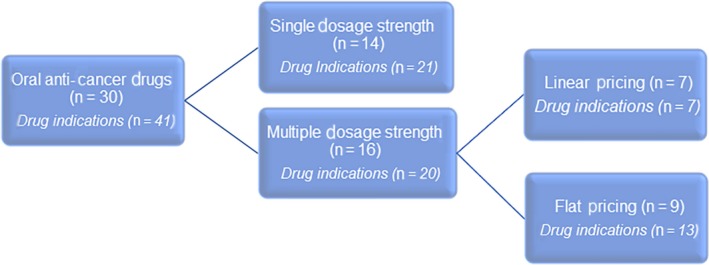
Oral anti‐cancer drugs reviewed

In total, 30 oral anti‐cancer drugs for use in 41 indications were included in our analysis (Figure 1). Lenvatinib was not included in the analysis due to a different pricing structure involving combination packages (Appendix S2). Sixty‐eight percent (28/41) of the drug indications were for the treatment of solid cancers and 32% (13/41) were for hematological cancers (Table 1). Sixty percent (18/30) of the drugs were in the form of a capsule and 40% (12/30) were available as a tablet. Forty‐seven percent (14/30) of drugs were available in a single dosage strength and 53% (16/30) had multiple dosage strengths; 7, 5, 3, and 1 drug(s) were available in 2, 3, 4, and 5 dosage strengths, respectively. Nearly half of the drugs used linear pricing (7/16) and the other half used linear pricing (9/16).

**Table 1:** Oral anti‐cancer drugs reviewed

Generic Name	Brand Name	Indication(s)	Dosage Form
Abiraterone	Zytiga	mCRPC	Tablet
Alectinib	Alecensaro	NSCLC	Capsule
Afatinib	Giotrif	Advanced NSCLC	Tablet
Axitinib	Inlyta	mRCC	Tablet
Bosutinib	Bosulif	CML	Tablet
Ceritinib	Zykadia	NSCLC	Capsule
Cobimetinib	Cotellic	Metastatic melanoma	Tablet
Crizotinib	Xalkori	First‐line ALK‐positive advanced or metastatic NSCLC	Tablet
Dabrafenib	Tafinlar	Metastatic melanoma	Capsule
Enzalutamide	Xtandi	1) mCRPC: previously received docetaxel; 2) mCRPC: asymptomatic/mildly symptomatic after failure of androgen deprivation therapy who have not received prior chemotherapy	Capsule
Everolimus	Afinitor	1) Advanced BC; 2) NET of gastrointestinal or lung origin; 3) pNET	Tablet
Ibrutinib	Imbruvica	1) CLL/SLL (previously treated); 2) CLL/SLL (previously untreated); 3) Mantle cell lymphoma; 4) Waldenstrom's macroglobulinemia	Capsule
Idelalisib	Zydelig	1) CLL; 2) Follicular lymphoma	Tablet
Ixazomib	Ninlaro	MM	Capsule
Lapatinib	Tykerb	mBC	Tablet
Lenalidomide	Revlimid	1) MM (newly diagnosed); 2) MM (maintenance)	Capsule
Olaparib	Lynparza	Ovarian cancer	Capsule
Osimertinib	Tagrisso	NSCLC	Tablet
Palbociclib	Ibrance	Advanced BC	Capsule
Pazopanib	Votrient	1) mRCC 2) STS	Tablet
Pomalidomide	Pomalyst	MM	Capsule
Ponatinib	Iclusig	CML/ALL	Tablet
Regorafenib	Stivarga	1) GIST 2) mCRC	Tablet
Ruxolitinib	Javaki	1) Myelofibrosis; 2) Polycythemia vera	Tablet
Sorafenib	Nexavar	mDTC	Tablet
Sunitinib	Sutent	pNET	Tablet
Trametinib	Mekinist	Metastatic melanoma	Tablet
Vandetanib	Caprelsa	Medullary thyroid cancer	Tablet
Vemurafenib	Zelboraf	Advanced melanoma	Tablet
Vismodegib	Erivedge	Advanced BCC	Capsule

Table 1 shows the 30 oral anti‐cancer drugs reviewed, their indication(s), and dosage forms.

Abbreviations: ALL, acute lymphoblastic leukemia; BC, breast cancer; BCC, basal cell carcinoma; CLL, chronic lymphocytic leukemia; CML, chronic myeloid leukemia; mBC, metastatic breast cancer; mCRC, metastatic colorectal cancer; mCRPC, metastatic castration‐resistant prostate cancer; mDTC, metastatic differentiated thyroid carcinoma; MM, multiple myeloma; mRCC, metastatic renal cell carcinoma; NSCLC, non‐small cell lung cancer; pNET, pancreatic neuroendocrine tumour; SLL, small lymphocytic leukemia; STS, soft tissue sarcoma.

### 3.2 Dose modifications

Table 2 shows the recommended starting dose and dose modifications for dose levels (−2 to +2) for each drug indication. Nearly all the drug indications (39/41) had a possible dose reduction except for idelalisib (both indications) and vismodegib. In contrast, only four drug indications had a possible dose escalation: axitinib, lenalidomide for maintenance in multiple myeloma, and ruxolitinib (both indications). Dose reduction to dose level −1 was possible in all drug indications using linear (7/7) and flat pricing (13/13), and 90% (19/21) of drug indications with a single dosage strength. Dose reduction to dose level −2 was possible in 86% (6/7) of drug indications using linear pricing, 77% (10/13) of flat pricing, and 90% (19/21) of single dosage strength.

**Table 2:** Dose level modifications for the oral anti‐cancer drugs reviewed

Drug	Indication	Dosage Strength(s) (mg)	Starting Dose	Dose Level −1	Dose Level −2	Dose Level +1	Dose Level +2
Abiraterone	mCRPC	250	1000 mg QD	750 mg QD	500 mg QD	N/A	N/A
Afatinib	NSCLC	20, 30, 40, 50	40 mg QD	30 mg QD	20 mg QD	N/A	N/A
Alectinib	NSCLC	150	600 mg BID	450 mg BID	300 mg BID	N/A	N/A
Axitinib	mRCC	1, 5	5 mg BID	3 mg BID	2 mg BID	7 mg BID	10 mg BID
Bosutinib	CML	100, 500	500 mg QD	400 mg QD	300 mg QD	N/A	N/A
Ceritinib	NSCLC	150	750 mg QD	600 mg QD	450 mg QD	N/A	N/A
Cobimetinib	Melanoma	20	60 mg QD for 21 days	40 mg QD for 21 days	20 mg QD for 21 days	N/A	N/A
Crizotinib	NSCLC	200, 250	250 mg BID	200 mg BID	250 mg QD	N/A	N/A
Dabrafenib	Melanoma	50, 75	150 mg BID	100 mg BID	75 mg BID	N/A	N/A
Enzalutamide	mCRPC (no previous docetaxel)	40	160 mg QD	120 mg QD	80 mg QD	N/A	N/A
Enzalutamide	mCRPC (previous docetaxel)	40	160 mg QD	120 mg QD	80 mg QD	N/A	N/A
Everolimus	Advanced BC	2.5, 5, 10	10 mg QD	5 mg QD	5 mg every other day	N/A	N/A
Everolimus	NET of gastrointestinal or lung origin	2.5, 5, 10	10 mg QD	5 mg QD	5 mg every other day	N/A	N/A
Everolimus	pNET	2.5, 5, 10	10 mg QD	5 mg QD	5 mg every other day	N/A	N/A
Ibrutinib	CLL/SLL (previously treated)	140	420 mg QD	280 mg QD	140 mg QD	N/A	N/A
Ibrutinib	CLL/SLL (previously untreated)	140	420 mg QD	280 mg QD	140 mg QD	N/A	N/A
Ibrutinib	Mantle cell lymphoma	140	560 mg QD	420 mg QD	280 mg QD	N/A	N/A
Ibrutinib	Waldenstrom's Macroglobulinemia	140	420 mg QD	280 mg QD	140 mg QD	N/A	N/A
Idelalisib	CLL	150	150 mg BID	100 mg BID	N/A	N/A	N/A
Idelalisib	Follicular lymphoma	150	150 mg BID	100 mg BID	N/A	N/A	N/A
Ixazomib	MM	2.3, 3, 4	4 mg QD on days 1, 8, 15	3 mg QD on days 1, 8, 15	2.3 mg QD on days 1, 8, 15	N/A	N/A
Lapatinib	mBC	250	1500 mg QD	1250 mg QD	1000 mg QD	N/A	N/A
Lenalidomide	MM (newly diagnosed MM)	5, 10, 15, 20^a^, 25	25 mg QD for 21 days	20 mg QD for 21 days	15 mg QD for 21 days	N/A	N/A
Lenalidomide	MM (maintenance MM)	5, 10, 15, 25	10 mg QD	5 mg QD	N/A	15 mg QD	N/A
Olaparib	Ovarian cancer	50	400 mg BID	200 mg BID	100 mg BID	N/A	N/A
Osimertinib	NSCLC	40, 80	80 mg QD	40 mg QD	N/A	N/A	N/A
Palbociclib	Advanced BC	75, 100, 125	125 mg QD for 21 days	100 mg QD for 21 days	75 mg QD for 21 days	N/A	N/A
Pazopanib	mRCC	200	800 mg QD	600 mg QD	400 mg QD	N/A	N/A
Pazopanib	STS	200	800 mg QD	600 mg QD	400 mg QD	N/A	N/A
Pomalidomide	MM	1, 2, 3, 4	4 mg QD for 21 days	3 mg QD for 21 days	2 mg QD for 21 days	N/A	N/A
Ponatinib	CML/ALL	15, 45	45 mg QD	30 mg QD	15 mg QD	N/A	N/A
Regorafenib	GIST	40	160 mg QD for 21 days	120 mg QD for 21 days	80 mg QD for 21 days	N/A	N/A
Regorafenib	mCRC	40	160 mg QD for 21 days	120 mg QD for 21 days	80 mg QD for 21 days	N/A	N/A
Ruxolitinib	Myelofibrosis	5, 10^a^ , 15, 20	20 mg BID^a^	15 mg BID^a^	10 mg BID^a^	25 mg BID^a^	N/A^a^
Ruxolitinib	Polycythemia vera	5, 10, 15, 20	10 mg BID	5 mg BID	N/A	15 mg BID	20 mg BID
Sorafenib	mDTC	200	400 mg BID	400 mg, 200 mg 12 hours apart daily	200 mg BID	N/A	N/A
Sunitinib	pNET	12.5, 25, 50	37.5 mg QD	25 mg QD	N/A	50 mg QD	N/A
Trametinib	Metastatic melanoma	0.5, 1, 2	2 mg QD	1.5 mg QD	1 mg QD	N/A	N/A
Vandetanib	Medullary thyroid cancer	100, 300	300 mg QD	200 mg QD	100 mg QD	N/A	N/A
Vemurafenib	Advanced melanoma	240	960 mg BID	720 mg BID	480 mg BID	N/A	N/A
Vismodegib	Advanced BCC	150	150 mg QD	N/A	N/A	N/A	N/A

Table 2 shows the pricing strategy, available drug strength(s), recommended starting dose for a 28‐day cycle unless specified, and dose level modifications (dose levels: −2 to +2) for the 41 drug indications analyzed.

Abbreviations: ALL, acute lymphoblastic leukemia; BC, breast cancer; BCC, basal cell carcinoma; BID, bidaily; CLL, chronic lymphocytic leukemia; CML, chronic myeloid leukemia; mBC, metastatic breast cancer; mCRC, metastatic colorectal cancer; mCRPC, metastatic castration‐resistant prostate cancer; mDTC, metastatic differentiated thyroid carcinoma; MM, multiple myeloma; mRCC, metastatic renal cell carcinoma; NSCLC, non‐small cell lung cancer; pNET, pancreatic neuroendocrine tumour; QD, once daily; SLL, small lymphocytic leukemia; STS, soft tissue sarcoma.

a Recommended starting dose and dose level modifications depend on platelet count.

### 3.3 Impact of dose reductions on drug costs

Table 3 shows the impact of dose reductions on the cost per mg and cost per 28‐day cycle. Dose reduction increased the mean cost per mg for drugs with linear pricing by 14.7% (n = 7 drugs, n = 7 indications, range: 0%‐50%) at dose level −1 and 17.2% (n = 6 drugs, n = 6 indications, range: 0%‐50%) at dose level −2. Dose reduction increased the mean cost per mg for drugs with flat pricing by 60.8% (n = 9 drugs, n = 13 indications, range: 18.8% to 100%) at dose level −1 and 99.1% (n = 8 drugs, n = 10 indications, range: 0%‐300%) at dose level −2. The cost per mg was significantly increased in drugs using flat pricing compared to linear pricing when dose reduction from dose level 0 to either level −1 (*P* = 0.010) or level −2 (*P* = 0.006) occurred. The number of drugs that had an increase in cost per mg when the standard dose is reduced to dose level −1 was significantly greater in drugs that used flat pricing compared to linear pricing (*P* = 0.007), but there was no significant difference with reduction to dose level −2 (*P* = 0.118).

**Table 3:** Impact of dose reductions on drug cost per mg and cost per cycle by pricing strategy

			Cost per mg ($/mg)	∆Cost per mg (%)		Cost per 28 days ($)	∆Cost per 28 days (%)	
Drug	Indication	Price Ratio	Dose Level 0	Dose Level −1	Dose Level −2	Dose Level 0	Dose Level −1	Dose Level −2
Linear Pricing (n = 7 drugs, n = 7 indications)								
Axitinib	mRCC	1	18.60	0%	0%	5,208	−15%	−57%
Bosutinib	CML	0.94	0.29	+25%	+25%	4,098	−33%	N/A
Dabrafenib	Metastatic melanoma	1	0.84	0%	0%	7,093	−25%	−50%
Ponatinib	CML/ALL	0.86	7.35	+28%	+28%	9,262	−15%	−57%
Sunitinib	pNET	1	5.05	0%	N/A	5,305	−33%	N/A
Trametinib	Metastatic melanoma	1	145.00	0%	0%	8,120	−25%	−50%
Vandetanib	Medullary thyroid cancer	0.75	0.65	+50%	+50%	5,460	0%	−50%
Flat Pricing (n = 9 drugs, n = 13 indications)								
Afatinib	Advanced NSCLC	0	2.00	+33%	+100%	2,240	0%	0%
Crizotinib	NSCLC	0	0.59	+25%	0%	8,214	0%	−50%
Everolimus	Advanced BC	0	18.60	+100%	100%	5,208	0%	−50%
Everolimus	NET of GI or lung	0	18.60	+100%	100%	5,208	0%	−50%
Everolimus	pNET	0	18.60	+100%	100%	5,208	0%	−50%
Ixazomib	MM	0	741.16	+33%	+74%	8,894	0%	0%
Lenalidomide	MM (maintenance)	0.12	36.10	+88%	N/A	10,108	−6%	N/A
Lenalidomide	MM (newly diagnosed)	0.25	16.96	+19%	+50%	11,872	−5%	−10%
Osimertinib	NSCLC	0	3.68	+100%	N/A	8,251	0%	N/A
Palbociclib	Advanced BC	0	2.38	+25%	+67%	8,333	0%	0%
Pomalidomide	MM	0	125.00	+33%	+100%	14,000	0%	0%
Ruxolitinib	Myelofibrosis (assumes 10 mg available)^a^	0	4.11	+33%	+100%	4,603	0%	0%
Ruxolitinib	Myelofibrosis (assumes 10 mg not available)	0	4.11	33%	300%	4,603	0.0%	100.0%
Ruxolitinib	Polycythemia vera	0	8.22	100%	N/A	4,603	0%	N/A
Single Dosage Strength (n = 14 drugs, n = 21 indications)								
Abiraterone	mCRPC	N/A	0.11	0%	0%	3,173	−25.0%	−50.0%
Alectinib	NSCLC	N/A	0.28	0%	0%	9,453	−25.0%	−50.0%
Ceritinib	NSCLC	N/A	0.45	0%	0%	9,446	−20.0%	−40.0%
Cobimetinib	Melanoma	N/A	6.00	0%	0%	10,080	−33.3%	−66.7%
Enzalutamide	mCRPC (no previous docetaxel)	N/A	0.71	0%	0%	3,174	−25.0%	−50.0%
Enzalutamide	mCRPC (previous docetaxel)	N/A	0.71	0%	0%	3,174	−25.0%	−50.0%
Ibrutinib	CLL/SLL (previously untreated)	N/A	0.65	0%	0%	7,615	−33.3%	−66.7%
Ibrutinib	CLL/SLL (previously treated)	N/A	0.65	0%	0%	7,615	−33.3%	−66.7%
Ibrutinib	Mantle cell lymphoma	N/A	0.65	0%	0%	10,153	−25.0%	−50.0%
Ibrutinib	Waldenstrom's macroglobulinemia	N/A	0.65	0%	0%	7,615	−33.3%	−66.7%
Idelalisib	CLL	N/A	0.57	N/A	N/A	4,780	N/A	N/A
Idelalisib	Follicular lymphoma	N/A	0.57	N/A	N/A	4,780	N/A	N/A
Lapatinib	mBC	N/A	0.09	0%	0%	3,948	−16.7%	−33.3%
Olaparib	Ovarian cancer	N/A	0.33	0%	0%	7,500	−50.0%	−75.0%
Pazopanib	STS	N/A	0.21	0%	0%	4,592	−25.0%	−50.0%
Pazopanib	mRCC	N/A	0.21	0%	0%	4,592	−25.0%	−50.0%
Regorafenib	GIST	N/A	1.86	0%	0%	8,316	−25.0%	−50.0%
Regorafenib	mCRC	N/A	1.82	0%	0%	8,133	−25.0%	−50.0%
Sorafenib	mDTC	N/A	0.23	0%	0%	5,227	−25.0%	−50.0%
Vemurafenib	Advanced melanoma	N/A	0.19	0%	0%	10,425	−25.0%	−50.0%
Vismodegib	Advanced BCC	N/A	1.96	N/A	N/A	8,238	N/A	N/A

Table 3 shows drug cost per mg ($/mg) at dose level 0 and the percent change in cost per mg (%) due to dose reductions (dose levels −1 and −2) with respect to dose level 0. It also shows the cost per 28‐day cycle ($) at dose level 0 and the percent change in cost per 28 days (%) due to dose reductions (dose levels −1 and −2) with respect to dose level 0.

Abbreviations: ALL, acute lymphoblastic leukemia; BC, breast cancer; BCC, basal cell carcinoma; BID, bidaily; CLL, chronic lymphocytic leukemia; CML, chronic myeloid leukemia; mBC, metastatic breast cancer; mCRC, metastatic colorectal cancer; mCRPC, metastatic castration‐resistant prostate cancer; mDTC, metastatic differentiated thyroid carcinoma; MM, multiple myeloma; mRCC, metastatic renal cell carcinoma; NSCLC, non‐small cell lung cancer; pNET, pancreatic neuroendocrine tumour; QD, once daily; SLL, small lymphocytic leukemia; STS, soft tissue sarcoma.

a Not included in main analysis.

**Figure 2:**
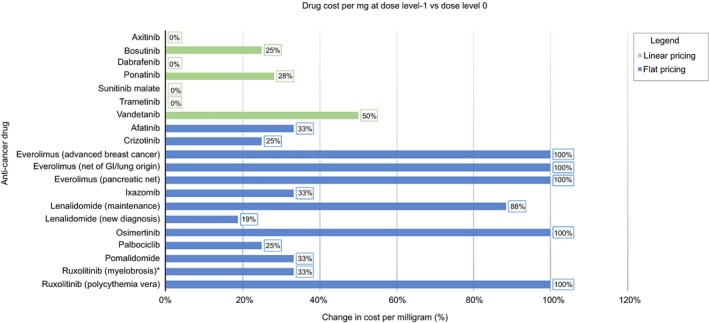
Impact of pricing strategy on cost per mg during dose reduction to dose level −1

**Figure 3:**
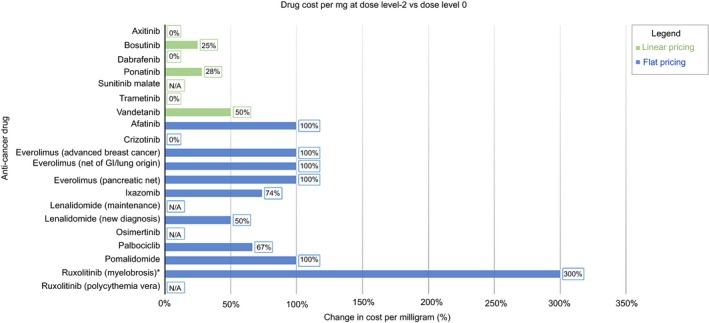
Impact of pricing strategy on cost per mg during dose reduction to dose level −2

Figure 2 and Figure 3 compares the percent change in cost per mg at dose level −1 and −2, respectively, for drugs using linear and flat pricing. In general, drugs using flat pricing had a greater increase in the cost per mg compared to those with linear pricing; half of the flat‐priced drugs (afatinib, everolimus, pomalidomide, ruxolitinib) doubled in cost per mg during dose reductions. Flat‐priced drugs that increased in cost at dose level −1 continued to increase at dose level −2, reaching a maximum of 100% with the exception of ruxolitinib for myelofibrosis, which reached 300%. With the 10 mg tablet of ruxolitinib now available, the percent increase in cost per mg decreased from 300% to 100% at dose level −2. This increasing trend was not seen in linear‐priced drugs. Instead, the percent increase in cost per mg remained the same with additional dose reduction.

The mean cost per 28‐day cycle was decreased for drugs with linear pricing by 20.9% (range: −40% to 0%) at dose level −1 and 48.7% (range: −60% to −25%) at dose level −2 and flat pricing by 0.8% (range: −5.8% to 0%) at dose level −1 and 11.0% (range: −50% to 100%) at dose level −2 compared to dose level 0 across all indications. The cost per cycle was significantly decreased in drugs with linear pricing compared to flat pricing when the standard dose is reduced to either dose level −1 (*P* = 0.005) and dose level −2 (*P* = 0.026). The number of drugs that had a decrease in cost per cycle was significantly greater in linear‐priced drugs compared to flat‐priced drugs when the standard dose is reduced to level −1 (*P* = 0.022) but there was no significant difference at dose level −2 (*P* = 0.093).

In general, drugs using linear pricing had a proportional decrease in the cost per cycle during dose reductions while those with flat pricing had minimal changes in costs despite using lower doses. A notable exception is the case of ruxolitinib for myelofibrosis, where dose reduction increased the cost by 100% at dose level −2. With the 10 mg tablet of ruxolitinib now available, the cost per cycle remained the same as the standard dose despite dose reductions. Finally, the cost per cycle was proportionally decreased for drugs with a single dosage strength.

### 3.4 Impact of dose escalations on drug costs

Table 4 shows the impact of dose escalations on the cost per mg and cost per 28‐day cycle. Axitinib, which uses linear pricing, showed no changes in the cost per mg when the dose was escalated. In contrast, drugs using flat pricing (n = 2 drugs used in 3 indications) had a mean decrease in the cost per mg by 0.9% (range: −33.3% to 60.0%) at dose level +1. Only ruxolitinib when used for polycythemia vera could be dose escalated to dose level +2, which resulted in a 50% decrease in the cost per mg. The cost per cycle of axitinib increased by 40.0% at dose level +1 and 100% at dose level +2. The drugs using flat pricing had variable changes in the cost per cycle when dose escalated. At dose level +1, the cost per cycle increase by 5.8% for lenalidomide and by 100% for ruxolitinib when used for idiopathic myelofibrosis. In contrast, ruxolitinib for polycythemia vera had no changes in the cost per cycle at dose level +1 and +2. Overall, the impact of pricing strategy on drugs during dose escalations could not be determined due to the limited number of drugs.

**Table 4:** Impact of dose escalations on drug cost per mg and cost per cycle by pricing strategy

			Cost per mg ($/mg)	∆Cost per mg (%)		Cost per 28 days ($)	∆Cost per 28 days (%)	
Drug	Indication	Price Ratio	Dose Level 0	Dose Level +1	Dose Level +2	Dose Level 0	Dose Level +1	Dose Level +2
Linear Pricing (n = 1 drug, n = 1 indication)								
Axitinib	mRCC	1	18.60	0.0%	0.0%	5,208	40%	100%
Flat Pricing (n = 2 drugs, n = 3 indications)								
Lenalidomide	MM (maintenance)	0.12	36.10	−29.5%	N/A	10,108	5.8%	N/A
Ruxolitinib	Myelofibrosis	0	4.11	60.0%	N/A	4,603	100%	N/A
Ruxolitinib	Polycythemia vera	0	8.22	−33.3%	−50.0%	4,603	0%	0%

Table 4 shows drug cost per mg ($/mg) at dose level 0 and the percent change in cost per mg (%) due to dose escalations (dose levels +1 and +2) with respect to dose level 0. It also shows the cost per 28‐day cycle ($) at dose level 0 and the percent change in cost per 28 days (%) due to dose escalations (dose levels +1 and +2) with respect to dose level 0.

Abbreviations: MM: multiple myeloma; mRCC: metastatic renal cell carcinoma.

## 4 DISCUSSION

Our analysis of the oral anti‐cancer drugs showed that pricing strategy affects costs during dose modifications. The manufacturers’ adopted the flat pricing strategy in slightly more than half of the drugs (56%) reviewed in this study. In general, dose reduction resulted in a significantly greater increase in the cost per mg for drugs using flat pricing (of up to 300%) compared to linear pricing. Despite using lower doses, the cost per cycle remained relatively the same for drugs with flat pricing, while it decreased proportionally for linear pricing. The impact of pricing strategy on dose escalations could not be determined because only a few drugs could be dose escalated. Overall, the flat pricing strategy is significantly more costly than linear pricing for oral anti‐cancer drugs.

A study of drugs with multiple dosages from the Ontario formulary, which does not include anti‐cancer medications, showed that 23% (17/73) used perfect flat pricing and 26% (19/73) monotonic pricing.^26^ In contrast to our findings, this study found that flat pricing resulted in lower expenditures, while monotonic pricing led to higher expenditures. This finding may be because most of the drugs in their study, including antihypertensive medications and antidepressants, are administered starting at a low dose and titrated upwards to achieve the minimum effective dose. The author suggested that flat pricing should be required to be listed in the formulary. Although flat pricing may have beneficial cost savings for drugs that require dose escalation and enables better predictability of expenditures, it is not ideal in the case of oral anti‐cancer medications, where dose reductions occur frequently. Payers would be spending the same amount of money for no additional value, despite getting a lower dose, if the drug had a flat price.

In the oncology setting, patients are usually started on the maximum tolerated dose of the anti‐cancer drug(s). If a severe toxicity develops, the dose could be reduced or discontinued. As such, dose reductions are very common with the use of anti‐cancer medications, while dose escalations are rare. A review of oral anti‐cancer agents with putative primary targets of VEGF and RET encompassing 66 clinical trials showed that approximately one‐third of patients required a dose reduction.^27^ In the real world, dose reductions may occur even more frequently compared to data in clinical trials since these patients are carefully selected and must meet stringent eligibility criteria. Certain groups of patients, including the elderly, who are more prone to require dose reductions may therefore be disadvantaged due to the flat pricing strategy which would cause them to incur additional costs.

This is the first study to our knowledge to explore the impact of pricing strategy on costs during dose modifications of oral anti‐cancer drugs. However, our study has several limitations that should be considered. First, this analysis was based on Canadian data that was publicly available through the CADTH website, which may not be reflective of anti‐cancer drug pricing practices in other countries. Although there may be variation in drug prices, the impact of pricing strategy on drug costs during dose modifications of anti‐cancer drugs should remain applicable. Furthermore, our analysis does not include confidential prices that may be negotiated independently, which may possibly result in price reductions that account for flat versus linear pricing. The protocols for dose modifications of oral anti‐cancer drugs can also vary considerably.^27^ Our study did not capture the costs of drug wastage associated with premature discontinuation of a package of drug when dose modification was required. Finally, current studies generally do not provide specific data concerning the number of patients undergoing each dose level modification and/or do not report the specific length of time that the modified dose was administered. Furthermore, the current economic models reviewed by the pCODR do not provide the option for modeling specific dose level modifications for a proportion of patients. As a result, we could only perform limited cost‐effectiveness analyses. We hypothesized that dose reduction would have minimal effect on the incremental cost‐effectiveness ratio (ICER) and budget of flat‐priced drugs, while the ICER and budget would decrease for linear‐priced drugs based on the drug cost per 28 days. However, dose modifications of drugs using flat pricing that require additional number of tablets or capsules, would increase the ICER and budget.

We were able to reanalyze two economic evaluations submitted to pCODR by modeling dose adjustments for all patients and the associated drug cost per tablet or mg at the modified dose level to highlight this issue. When we reanalyzed the cost‐effectiveness analysis of ruxolitinib for myelofibrosis, assuming a dose reduction from 15 mg (one 15 mg tablet) to 10 mg (two 5 mg tablets) bi‐daily, the ICER increased by 157%. We reanalyzed the economic model for everolimus for advanced breast cancer, assuming a dose reduction from 10 mg (one 10 mg tablet) to 7.5 mg (combining one 2.5 mg tablet and one 5 mg tablet); the ICER increased by 84% and the 3‐year total budget increased by 100%. If dose reductions are required, drugs using flat pricing will likely have financial wastage compared to linear pricing, with the potential to increase associated ICERs and budget impacts. We recommend that future economic evaluation guidelines include recommendations on explicit modeling that account for different types of pricing strategies and the proportion of patients that require dose modifications.

## 5 CONCLUSION

The impact of pricing strategy on the costs of oral anti‐cancer drugs remains underrecognized in the literature, but it has potentially significant cost implications due to the high cost, extensive utilization, and the frequent occurrence of dose reduction. Future studies delineating the dose level modifications required by what proportion of patients and the development of economic models accounting for dose level modifications will assist in calculating the true costs of these drugs. Flat pricing of oral anti‐cancer drugs can lead to wastage if payers are spending the same amount of money on drugs despite purchasing lower doses. It can also unjustly discriminate against groups that are more prone to require dose reduction. Policymakers should consider discouraging the use of flat pricing for oral anti‐cancer drugs.

## CONFLICTS OF INTEREST

At the time of initiating this research, Mona Sabharwal was the inaugural Executive Director of the pan‐Canadian Oncology Drug Review. She is currently affiliated with Rexall Canada, a national pharmacy services provider. The remaining authors have reported no potential conflicts of interest.

## AUTHOR CONTRIBUTIONS

Judy Truong: conceptualization, data curation, methodology, formal analysis, and writing – original draft and editing. Dr Kelvin K.W. Chan: conceptualization, supervision, resources, methodology, formal analysis, writing – review and editing. Helen Mai: writing – review and editing. Alexandra Chambers: writing – review and editing. Dr Mona Sabharwal: writing – review and editing. Dr Maureen E. Trudeau: writing – review and editing. Dr Matthew C. Cheung: conceptualization, supervision, resources, methodology, formal analysis, writing – review and editing.
