# Supplementary material for: The impact of pricing strategy on the costs of oral anti‐cancer drugs
Source: Cancer Med. 2019 May 27;8(8):3770–81. doi: 10.1002/cam4.2269 (PMC6639183; doi:10.1002/cam4.2269)
Supplement: Supplementary file 1 [file CAM4-8-3770-s001.docx]

**Appendix 1: Sample price ratio calculations**

i) Flat pricing

1. Afatinib is available in 20, 30, 40, and 50 mg tablets. All tablets are priced at $80.

Price ratio = $\frac{(\$80-\$80)/(50 mg-20 mg)}{(\frac{\$80}{20 mg})}$

= 0

Thus, afatinib has perfect flat pricing since the price ratio is 0.

1. Lenalidomide is available in 5, 10, 15, 20, and 25 mg capsules priced at $340, $361, $382, $403, and $424, respectively.

Price ratio = $\frac{(\$424-\$340)/(25 mg-5 mg)}{(\frac{\$340}{5 mg})}$

= 0.25

Thus, lenalidomide has a flat pricing structure since the price ratio is less than 0.33.

ii) Linear pricing

1. Axitinib is available in 1 and 5 mg tablets priced at $18.60 and $93.00, respectively.

Price ratio = $\frac{(\$93.00-\$18.60)/(5 mg-1 mg)}{(\frac{\$18.60}{1 mg})}$

= 1

Thus, axitinib has perfect linear pricing since the price ratio is 1.

1. Vandetanib is available in 100 and 300 mg tablets priced at $97.50 and $195.00, respectively.

Price ratio = $\frac{(\$195.00-\$97.50)/(300 mg-100 mg)}{(\frac{\$97.50}{100 mg})}$

= 0.75

Thus, vandetanib has a linear pricing since the price ratio is greater than 0.66.

**Appendix 2: Lenvatinib pricing**

Lenvatinib has a unique pricing strategy compared to the drugs reviewed. Lenvatinib is available in 4 mg ($38.78) and 10 mg ($71.64) capsules. The recommended dose for dose level 0 is 24 mg daily, dose level -1 is 20 mg daily, and dose level -2 is 14 mg daily. It can be purchased in pre-packaged combinations of 24 mg ($220.84), 20 mg ($165.64), and 14 mg ($110.42). Although the 24 mg and 20 mg pre-packaged versions of lenvatinib are more convenient, self-combining the 10 mg and 14 mg packages to make the 24 mg dose (dose level 0) or obtaining 2 packages of the 10 mg capsules to make the 20 mg dose ($143.64) enables cost savings. Its pricing structure also influences the cost per mg and cost per 28 days when dose reduced. There is a decrease in the cost per mg of the pre-packaged version by 10.5% at dose level -1 and 14.3% at dose level -2 compared to dose level 0. The cost per 28 days of the pre-package version decrease by 25.0% at dose level -1 and 50.0% at dose level -2 compared to dose level 0. Using self-combined packages, the cost per mg decreases by 6% at dose level -1 and increases by 4% at dose level -2, while the cost per 28 days decreases by 21% at dose level -1 and 39% at dose level -2.
